# Additomultiplicative Cascades Govern Multifractal Scaling Reliability Across Cardiac, Financial, and Climate Systems

**DOI:** 10.3390/e28030359

**Published:** 2026-03-22

**Authors:** Madhur Mangalam, Eiichi Watanabe, Ken Kiyono

**Affiliations:** 1 Department of Biomechanics, University of Nebraska at Omaha, Omaha, NE 68182, USA; 2 Department of Cardiology, Fujita Health University School of Medicine, Toyoake 470-1192, Japan; 3 Graduate School of Engineering Science, University of Osaka, Toyonaka 560-8531, Japan

**Keywords:** cascade dynamics, fluctuations, fractal, interactivity, lognormal, multifractal, surrogation

## Abstract

The generative mechanisms underlying multifractal scaling in complex systems remain a fundamental unsolved problem, limiting our ability to distinguish healthy from pathological dynamics, predict system failures, or understand how scale-invariant organization emerges across vastly different physical domains. We resolve this challenge by introducing threshold sensitivity analysis—an extension of Chhabra–Jensen’s direct method—as a framework that classifies cascade types by examining how scaling reliability varies across moment orders *q*. Different *q* values systematically probe weak fluctuations (negative *q*) versus strong fluctuations (positive *q*), and the coefficient of determination ($r^{2}$) of partition function regressions quantifies scaling reliability at each *q*. Analyzing $r^{2}\left(q\right)$ patterns in 280 cardiac recordings (healthy controls through fatal heart failure), 200 financial time series (global equity markets and currencies, 2000–2025), and 80 climate stations (tropical to continental zones, 2000–2025), we discover a universal diagnostic signature: symmetric expansion of valid scaling behavior under relaxed $r^{2}$ thresholds, spanning both weak and strong fluctuations. This threshold sensitivity fingerprint—predicted by synthetic cascade simulations but never before validated empirically—uniquely identifies additomultiplicative cascades, hybrid processes that randomly alternate between additive stabilization and multiplicative amplification. Critically, this symmetric signature persists universally across domains: cardiac dynamics maintain consistent patterns across health and disease states, financial markets show varying robustness across asset classes (currencies more variable than US equities) while preserving a hybrid structure, and climate systems exhibit geographical variations (subtropical/continental stronger than tropical) without altering fundamental cascade type. These findings suggest that additomultiplicative organization is a unifying feature of complex adaptive systems, offering a resolution to decades of debate between additive and multiplicative models. The $r^{2}\left(q\right)$ profiling provides a mechanistic diagnostic capable of detecting early dysfunction, assessing system resilience, and revealing how environmental constraints shape—but do not determine—the fundamental principles governing multifractal complexity.

## 1. Introduction

Multifractal scaling represents one of nature’s most profound organizational principles, manifesting across physiological regulation, financial markets, and climate dynamics [1,2,3,4,5,6,7]. Yet this universality poses a fundamental paradox: if diverse systems exhibit similar scale-invariant signatures, what distinguishes healthy from pathological dynamics? What determines whether a financial market will maintain stability or collapse into crisis? How do climate systems balance gradual variations with extreme events? The defining characteristic of multifractal systems—maintaining coherent scaling laws across both weak fluctuations (small deviations from typical behavior) and strong fluctuations (large, rare events)—captures the duality between fine-grained control and adaptive bursts. Despite extensive empirical documentation, the generative mechanisms sustaining this organization remain unresolved, limiting our ability to model, diagnose, or predict system behavior.

Recent computational investigations using synthetic random binomial cascade simulations have identified three distinct generative mechanisms [8,9,10,11,12]. These models partition probability mass hierarchically across scales, with each parent cell redistributing its probability to offspring cells through specific mathematical operations. The redistribution involves random weights *W* that introduce variability at each hierarchical level, modulating how probability flows from coarse to fine scales. Cascade types differ fundamentally in how they apply these weights: additive cascades employ linear superposition ($p_{\mathrm{child}}=p_{\mathrm{parent}}+W$), multiplicative cascades use proportional scaling ($p_{\mathrm{child}}=p_{\mathrm{parent}}\times W$), while additomultiplicative cascades randomly alternate between these operations ($p_{\mathrm{child}}=p_{\mathrm{parent}}\;\Theta \;W$, where Θ∈{+,×} is independently selected for each offspring cell and *W* represents random perturbations). This dual mechanism in additomultiplicative cascades maintains baseline variability through additive stability while enabling adaptive amplification through multiplicative scaling—creating the mathematical conditions necessary for robust multifractal scaling across both weak and strong fluctuations.

**Figure 1:**
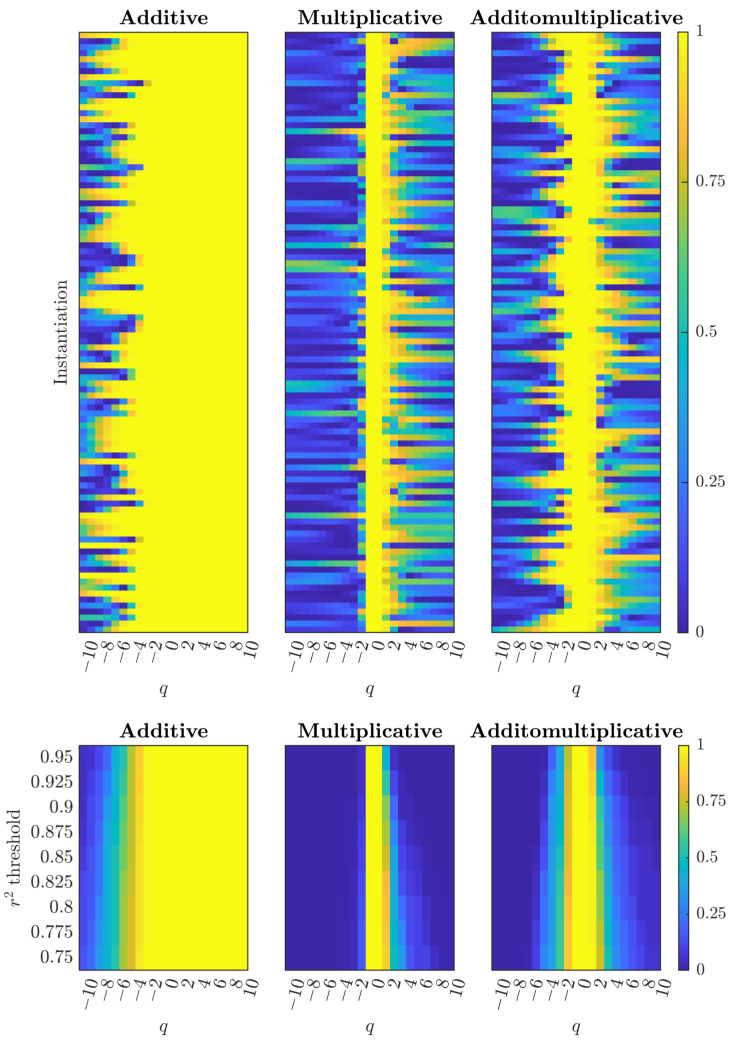
Theoretical predictions from synthetic cascade simulations establish distinct $r^{2}$ reliability signatures that distinguish generative mechanisms underlying multifractal scaling. **Top:** Representative $r^{2}$ heatmaps showing coefficient of determination patterns across moment orders q∈[−10,10] for n=100 individual synthetic cascade realizations: additive (**left**), multiplicative (**center**), and additomultiplicative (**right**) processes. Synthetic cascades were generated through 15 generations of hierarchical binary subdivision where parent probability units redistribute to offspring using additive ($p_{\mathrm{child}}=p_{\mathrm{parent}}+W$), multiplicative ($p_{\mathrm{child}}=p_{\mathrm{parent}}\times W$), or additomultiplicative ($p_{\mathrm{child}}=p_{\mathrm{parent}}\;\Theta \;W$) operations, where *W* represents Gaussian noise (μ=1, σ=1) and Θ is independently and randomly selected from {+,×} for each offspring cell. **Bottom:** Proportion of synthetic simulations achieving each $r^{2}$ threshold reveals characteristic cascade signatures for additive (**left**), multiplicative (**center**), and additomultiplicative (**right**) processes: additive cascades exhibit asymmetric reliability with systematic degradation for weak fluctuations (q<0), multiplicative cascades show narrow reliability zones centered near q∈[−1,1], while additomultiplicative cascades maintain symmetric expansion of valid scaling behavior across a broader range of *q* values. These theoretical patterns provide the framework for identifying cascade types in real-world complex systems.

The reliability of multifractal scaling can be quantified through the coefficient of determination ($r^{2}$) of the linear regressions used to estimate partition function scaling exponents in the Chhabra–Jensen direct method [13,14]. This method partitions time series into segments at multiple scales and computes probability measures within each segment, then examines how these measures scale across segment sizes through log–log regressions. The framework systematically varies moment orders *q*: negative *q* values emphasize weak fluctuations (small, frequent variations) while positive *q* values emphasize strong fluctuations (large, rare events). This multi-*q* approach captures the multiple scaling relationships defining multifractality [15], with $r^{2}$ values quantifying how well the data conform to power-law scaling at each *q*. Higher $r^{2}$ values indicate more consistent, predictable scaling behavior across scales. Recent systematic comparison of cascade structures across diverse noise environments revealed a striking pattern: only additomultiplicative processes maintain consistently high $r^{2}$ values across all *q* values, generating broad symmetric spectra through their unique capacity to couple weak and strong fluctuations [12]. Additive cascades show asymmetric degradation favoring strong fluctuations, multiplicative cascades exhibit narrow reliability zones, while additomultiplicative cascades maintain symmetric expansion of valid scaling behavior across a broader range of *q* values (Figure 1).

These simulation-based findings reveal a diagnostic opportunity. Traditional multifractal analysis applies strict $r^{2}$ thresholds to filter scaling relationships [13,14], discarding potentially informative behavior. However, the cascade-specific patterns reported by [12] suggest a different strategy: systematically analyzing how $r^{2}$ patterns change across the complete set of moment orders and under varying threshold constraints should reveal the underlying generative mechanisms. This threshold sensitivity analysis transforms $r^{2}\left(q\right)$ from a quality control filter into a mechanistic classifier capable of distinguishing cascade types in empirical data.

A critical insight underlies our threshold sensitivity approach: additomultiplicative systems generate scaling structure across the full spectrum of fluctuation intensities, but the reliability of this structure varies systematically. At extreme moment orders (large |q|), both the additive and multiplicative components continue to operate, but generate noisier scaling relationships than at moderate moment orders. Strict $r^{2}$ thresholds detect only the highest-quality scaling (typically around q≈0), while progressively relaxing thresholds reveals the broader extent of scaling structure extending toward both weak fluctuations (negative *q*) and strong fluctuations (positive *q*)—structure that was always present but too noisy to meet strict reliability criteria. This symmetric expansion under relaxed thresholds is the diagnostic signature of additomultiplicative organization: if both additive (baseline) and multiplicative (adaptive) components persist, then lowering reliability standards should reveal scaling relationships at both extremes of the *q*-spectrum. In contrast, purely additive systems would show asymmetric expansion favoring positive *q*, while purely multiplicative systems would show minimal expansion. By examining how the range of valid moment orders expands as thresholds relax, we can determine whether a system employs additomultiplicative coupling or fundamentally different organizational principles.

It is critical to emphasize that threshold sensitivity analysis is *not* a modification of the Chhabra–Jensen direct method [13] itself, but rather a novel interpretive framework that transforms how coefficient of determination values are used. Traditional applications of the Chhabra–Jensen direct method [13] treat $r^{2}$ thresholds as binary quality control filters: moment orders meeting strict criteria (e.g., $r^{2}>0.95$) are retained for multifractal spectrum construction, while those failing are discarded as unreliable. Our approach fundamentally reframes this usage by examining $r^{2}\left(q\right)$ patterns themselves as diagnostic signatures. Rather than filtering out low-$r^{2}$ regions, we systematically analyze *how* the range of valid moment orders expands as threshold constraints are progressively relaxed from strict ($r^{2}>0.95$) to moderate ($r^{2}>0.85$) levels. This expansion pattern—symmetric across both weak and strong fluctuations for additomultiplicative cascades, asymmetric for additive cascades, constrained for multiplicative cascades—reveals the underlying generative mechanism. The Chhabra–Jensen method provides the computational framework for obtaining $r^{2}$ values; threshold sensitivity analysis provides the mechanistic framework for interpreting those values as cascade fingerprints.

The critical question remains: do real-world complex systems exhibit these theoretical signatures? Despite compelling computational predictions, the biological and physical relevance of the additomultiplicative cascade model remains untested. This validation gap limits our ability to translate theoretical insights into practical applications. Without knowing which cascade type governs a system, we cannot distinguish healthy from pathological physiological regulation (does heart failure represent compromised integration of baseline regulation and burst responses, or collapse into unstable amplification cascades?), assess financial market resilience (do currency fluctuations reflect balanced price discovery mechanisms or panic-driven volatility amplification?), or understand climate system vulnerability (does reduced temperature variability indicate weakened coupling between steady-state dynamics and extreme events, or transition toward damped fluctuations?). Identifying cascade organization in empirical data would enable mechanistic diagnosis of system state, early detection of pathological transitions, and physics-based prediction of failure modes.

Threshold sensitivity analysis addresses a mechanistic classification problem that is fundamentally distinct from standard time series analysis tasks such as forecasting, anomaly detection, trend extraction, or noise smoothing. Our framework identifies the *generative processes* underlying observed multifractal scaling: determining whether a complex system employs additive cascades (linear superposition), multiplicative cascades (proportional amplification), or additomultiplicative cascades (hybrid mechanisms randomly alternating between both operations). This mechanistic classification enables three practical applications: (*i*) system state diagnosis—identifying whether observed dynamics conform to expected cascade organization for healthy or stable states (e.g., detecting pathological transitions in cardiac regulation before conventional clinical markers deteriorate, or identifying regime shifts in financial markets); (*ii*) model selection and constraint—knowing the cascade type constrains the mathematical framework appropriate for subsequent modeling, forecasting, or control tasks (systems with confirmed additomultiplicative organization require hybrid models incorporating both additive and multiplicative components rather than single-mechanism approaches); and (*iii*) noise separation from intrinsic structure—threshold sensitivity patterns distinguish scaling relationships intrinsic to the system (which expand symmetrically under relaxed thresholds) from measurement artifacts or environmental noise (which show irregular, asymmetric degradation), enabling more accurate multifractal spectrum estimation. The framework’s effectiveness is evaluated through discriminative power—the ability to correctly classify cascade types in controlled synthetic benchmarks and maintain consistent classification across independent empirical realizations.

We address this gap through a comprehensive analysis of three domains representing fundamental classes of complex systems. Each exhibits well-documented multifractal scaling yet arises from fundamentally different physical processes: cardiac dynamics emerge from coupled neural and mechanical feedback loops, financial markets from distributed agent interactions and information propagation, and climate systems from thermodynamic energy cascades across atmospheric scales. This diversity allows testing whether additomultiplicative organization represents a more unifying generative principle.

Our previous work established the $r^{2}\left(q\right)$ diagnostic framework using synthetic cascades and provided preliminary evidence in heart rate variability data [12]. Here, we extend this framework to systematic empirical validation across three independent physical domains—cardiac, financial, and climate systems—to test whether additomultiplicative organization represents a universal organizing principle rather than a domain-specific phenomenon. The present study moves beyond theoretical predictions to comprehensive cross-domain validation, examining whether the threshold sensitivity signatures predicted by synthetic cascades manifest consistently across fundamentally different physical processes that may share common cascade-level generative organization.

**Cardiac dynamics** (n=280 participants: healthy controls, heart failure survivors, nonsurvivors). Heart rate variability reflects the balance between sympathetic control (generating burst responses) and parasympathetic control (maintaining baseline regulation) [16,17,18,19]. If healthy cardiac regulation exhibits additomultiplicative organization, we should observe symmetric $r^{2}\left(q\right)$ profiles with high reliability across both weak fluctuations (parasympathetic fine-tuning) and strong fluctuations (sympathetic bursts). We examine whether heart failure alters threshold sensitivity patterns or preserves additomultiplicative organization despite pathological changes.

**Financial markets** (n=200 time series: global equity indices and currencies, 2000–2025). Volatility clustering and fat-tailed returns suggest cascade processes coupling incremental price adjustments with amplified volatility events [20,21,22]. If markets exhibit additomultiplicative organization, all asset classes should show symmetric threshold expansion despite varying robustness, with mature markets (US equities) exhibiting tighter organization than globally exposed systems (currencies).

**Climate systems** (n=80 stations: tropical, subtropical, temperate, continental zones, 2000–2025). Daily temperature fluctuations reflect coupling between gradual diurnal cycles and episodic weather events driven by synoptic-scale processes [23,24,25,26,27]. If additomultiplicative organization is universal, geographic diversity should modulate cascade robustness (tropical stability versus temperate variability versus continental extremes) while preserving symmetric threshold signatures across all climate zones.

Our objectives are to: (*i*) provide empirical validation of additomultiplicative cascade organization in real-world systems through systematic $r^{2}$ threshold sensitivity analysis, (*ii*) establish that the pattern of expansion under progressively relaxed $r^{2}$ thresholds distinguishes cascade types—symmetric expansion across both weak and strong fluctuations indicates additomultiplicative organization, while asymmetric patterns indicate additive or multiplicative organization—with the proportion of systems meeting each threshold quantifying organizational robustness, (*iii*) demonstrate universal additomultiplicative organization across cardiac, financial, and climate systems, and (*iv*) introduce $r^{2}\left(q\right)$ profiling as a mechanistic diagnostic framework that transcends traditional descriptive multifractal analysis.

## 2. Methods

### 2.1. Empirical Datasets

#### 2.1.1. Cardiac Dynamics

We analyzed RR interval time series from well-established cardiovascular cohorts with clearly defined clinical outcomes [28,29]. While previous work established multifractal properties of these datasets [29], the present study introduces systematic $r^{2}$ threshold sensitivity analysis to identify underlying cascade organization. The dataset comprised n=280 participants: healthy controls (n=172, mean±s.d. age: 48±18 years, 25 women, no cardiovascular disease history), heart failure survivors (n=69, 64±15 years, 27 women, no incidence within 33±17 months of follow-up period), and heart failure nonsurvivors (n=39, 70±14 years, 20 women, deceased within 33±17 months of follow-up period). The majority of deaths (34/39) were cardiac-related, including death from progressive heart failure (n=23), sudden death (n=10), and acute myocardial infarction (n=1); the remaining five patients died of sepsis (n=1), pneumonia (n=3), and stroke (n=1). All RR interval recordings were obtained from 24-h Holter monitoring, with preprocessing including automatic artifact detection and manual verification, followed by cubic spline interpolation to ensure uniform temporal sampling at 2 Hz. For multifractal analysis, time series were standardized to N=16,384 data points (approximately 2.3 h of continuous recording) to ensure consistent scale ranges across all participants and enable direct comparison with synthetic cascade benchmarks.

#### 2.1.2. Financial Markets

We obtained daily closing price data from major stock indices and currency exchange rates spanning July 2000 to June 2025, downloaded from https://www.finance.yahoo.com. The dataset included: S&P 500 (n=50 stocks), NASDAQ (n=50 stocks), Nikkei 225 (n=50 stocks), and major currency pairs (n=50 currency pairs). Each time series comprised the full 25-year period (mean±s.d. length: S&P 500, 6101±336 days; NASDAQ, 6102±330 days; Nikkei 225, 6104±652 days; currency pairs, 5805±303 days) to ensure sufficient length for multifractal estimation and systematic comparison across different financial instruments. All price data were converted to logarithmic returns $r_{t}=\ln\left(P_{t}/P_{t-1}\right)$ prior to multifractal analysis.

#### 2.1.3. Climate Systems

Daily temperature records were obtained from 80 diverse geographical locations (20 stations per climate zone) spanning Jan 2000 to Dec 2024 from www.visualcrossing.com. Stations were categorized into four distinct climate zones based on Köppen classification: tropical (consistent high temperatures with minimal seasonal variation), subtropical (warm temperatures with moderate seasonal cycles), temperate (pronounced seasonal temperature variations), and continental (extreme seasonal temperature ranges with rapid transitions). Daily maximum temperature in Fahrenheit was extracted for each station to capture the primary thermal variability signal while minimizing measurement noise associated with nighttime cooling processes. Each time series comprised the full 25-year period (mean±s.d. length: tropical, 9042±398 days; subtropical, 8037±895 days; temperate, 8494±871 days; continental, 8494±1045 days) to ensure sufficient length for robust multifractal estimation while maintaining consistent temporal coverage for reliable scaling analysis.

### 2.2. Multifractal Analysis Framework

#### Chhabra–Jensen Methodology

We employed the direct approach developed by Chhabra and Jensen [13] for all multifractal analyses, implemented with standardized parameters to ensure cross-domain comparability. This method quantifies multifractal complexity through systematic examination of scaling relationships across multiple scales without relying on distributional assumptions required by variance-based techniques such as the Multifractal Detrended Fluctuation Analysis (MF-DFA) [30,31] and the Wavelet Transform Modulus Maxima (WTMM) method [32].

The approach partitions each time series x(t) of length *T* into nonoverlapping segments of increasing size *n*, with scales selected as powers of 2: n∈{4,8,16,32,…} up to $n_{\max}=\left\lfloor T/8\right\rfloor$ to ensure adequate statistics with a minimum of 8 segments per scale. The maximum scale was determined by $n_{\max}=2^{\left\lfloor \log_{2}\left(\left\lfloor T/8\right\rfloor \right)\right\rfloor }$ to maintain power-of-2 scaling increments. For each scale *n*, the probability mass $P_{v}\left(n\right)$ is computed within each *v*-th segment ($v=1,2,\ldots ,N_{n}$ where $N_{n}=\left\lfloor T/n\right\rfloor$):

(1) $$P_{v}\left(n\right)=\frac{\sum_{k=(v-1)\;n+1}^{v\cdot n}\left|x\left(k\right)\right|}{\sum_{t=1}^{T}\left|x\left(t\right)\right|}.$$

The absolute values ensure positive probability measures while preserving multifractal structure. Probability measures are transformed into weighted distributions using moment parameter q∈[−10,10] with increment Δq=1:

(2) $$\mu_{v}\left(q,n\right)=\frac{{\left[P_{v}\left(n\right)\right]}^{q}}{\sum_{j=1}^{N_{n}}{\left[P_{j}\left(n\right)\right]}^{q}}.$$

The singularity strength α(q) and Hausdorff dimension f(q) are estimated through linear regression of the log–log relationships:

(3) $$\begin{array}{cc} \alpha (q) & =\lim_{n\to 0}\frac{1}{\ln n}\sum_{v=1}^{N_{n}}\mu_{v}\left(q,n\right)\ln P_{v}\left(n\right), \end{array}$$

(4) $$\begin{array}{cc} f(q) & =\lim_{n\to 0}\frac{1}{\ln n}\sum_{v=1}^{N_{n}}\mu_{v}\left(q,n\right)\ln\mu_{v}\left(q,n\right). \end{array}$$

Traditional multifractal analysis applies $r^{2}$ thresholds (typically $r^{2}>0.95$) to select only those moment orders *q* where linear scaling relationships meet stringent reliability criteria, using these filtered points to construct the multifractal spectrum {f(q),α(q)}. This approach discards potentially informative scaling behavior at moment orders where linearity is compromised. In contrast, we systematically analyze $r^{2}$ values across the complete moment order spectrum q∈[−10,10] without pre-filtering, examining how the reliability of linear fits for f(q) estimation varies with fluctuation intensity. While α(q) estimation is robust to moderate deviations from linearity, f(q) estimation is more sensitive to scaling relationship reliability, making $r^{2}$ constraints particularly relevant for the Hausdorff dimension component of the multifractal spectrum. By examining $r^{2}\left(q\right)$ patterns directly rather than applying them as exclusion criteria, we reveal the full landscape of scaling behavior across weak and strong fluctuations and identify systematic variations that reflect underlying generative mechanisms.

All analyses were implemented in MATLAB R2024a (MathWorks, Inc., Natick, MA, USA) using custom scripts validated against synthetic cascade benchmarks.

## 3. Results

### 3.1. Synthetic Cascade Simulations Establish Diagnostic Signatures

Before examining threshold sensitivity in empirical data, we first re-establish the expected patterns for each cascade type through synthetic binomial cascade simulations [12] (Figure 1). These simulations generate 100 instantiations of each cascade type and display their $r^{2}$ values across moment orders q∈[−10,10] as heatmaps, revealing how different generative mechanisms produce distinct scaling reliability patterns.

Additive cascades show a striking rightward bias: high $r^{2}$ values (yellow regions) dominate across positive moment orders (q>0), indicating robust scaling for strong fluctuations. However, at large negative moment orders (q<0, probing weak fluctuations), $r^{2}$ values degrade substantially, with most instantiations showing poor scaling reliability (blue regions). This asymmetry reflects the mathematical structure of additive cascades: linear superposition operations maintain consistent scaling for large-amplitude events while introducing noise that disrupts scaling precision for small-amplitude fluctuations.

Multiplicative cascades display the opposite pattern: high $r^{2}$ values concentrate around q≈0 in a narrow band, with rapid degradation toward both negative and positive extreme moment orders. This restriction arises because multiplicative operations (proportional scaling) generate tight scaling relationships around moderate fluctuations but introduce increasing variability as the analysis focuses on the distribution tails—both weak and strong fluctuations show degraded scaling precision.

Additomultiplicative cascades exhibit a distinctive symmetric pattern: high $r^{2}$ values (yellow regions) extend broadly across both negative and positive moment orders, forming a roughly symmetric distribution centered around q≈0. This breadth reflects the hybrid mechanism: random alternation between additive operations (maintaining baseline structure) and multiplicative operations (enabling adaptive scaling) produces reliable scaling relationships across the full spectrum of fluctuation intensities. Some instantiations show reduced $r^{2}$ at extreme moment orders, but the overall pattern remains symmetric—neither weak nor strong fluctuations are preferentially degraded.

The threshold sensitivity analysis (Figure 1, *bottom panels*) quantifies these organizational signatures by showing the proportion of simulations achieving each $r^{2}$ threshold across *q* values. Additive cascades maintain high proportions (>0.90) across all positive *q* values even under strict thresholds ($r^{2}>0.95$), but show complete failure at negative *q* values—the proportion achieving threshold remains low regardless of how much constraints are relaxed. This persistent asymmetry confirms that additive organization fundamentally cannot generate reliable scaling for weak fluctuations. Multiplicative cascades show minimal threshold dependence: high proportions are restricted to a narrow band around q∈[−2,2] at most threshold levels, with steep degradation toward positive *q* values. Relaxing thresholds produces minimal expansion toward positive *q* values, confirming that multiplicative organization cannot extend reliable scaling beyond moderate fluctuation intensities. Additomultiplicative cascades show symmetric threshold expansion: under strict constraints ($r^{2}>0.95$), high proportions concentrate around q∈[−2,2]. As thresholds progressively relax to $r^{2}>0.85$ and below, high proportions extend symmetrically toward both negative *q* (weak fluctuations) and positive *q* (strong fluctuations), revealing that both additive and multiplicative components generate detectable scaling structure across the full spectrum. This symmetric expansion is the definitive diagnostic signature: only additomultiplicative organization reveals scaling structure at both extremes when thresholds are relaxed—the structure was always present but too noisy to meet strict reliability criteria, confirming the simultaneous presence of additive and multiplicative interactions across scales.

### 3.2. Cardiac Dynamics Reveal Additomultiplicative Organization Across Health and Disease States

**Figure 2:**
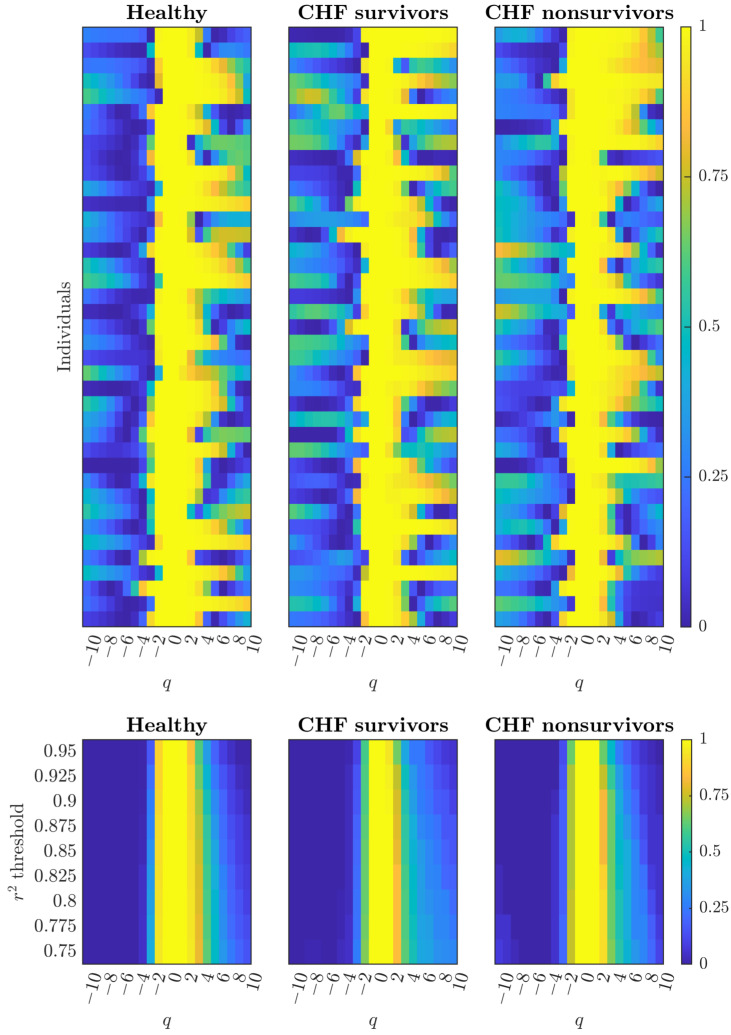
Cardiac RR interval dynamics across health and disease states show similar additomultiplicative threshold sensitivity patterns. **Top:** $r^{2}$ heatmaps across moment orders q∈[−10,10] for healthy controls (n=172), CHF survivors (n=69), and CHF nonsurvivors (n=39). Each row is an individual; each column is a *q*-value. Higher $r^{2}$ values (yellow) indicate stronger linear fits in the Chhabra–Jensen direct method [13]. All groups show peak reliability around q≈0 with degradation toward extremes, resembling additomultiplicative cascades. *Note:* 39 randomly selected individuals per group shown. **Bottom:** Proportion achieving each $r^{2}$ threshold shows similar patterns across groups: all concentrate around q∈[−2,3] with slight rightward bias. Symmetric expansion across negative *q* (weak fluctuations, parasympathetic) and positive *q* (strong fluctuations, sympathetic) confirms additomultiplicative organization across health and disease states.

Analysis of cardiac RR interval dynamics across 280 participants (172 healthy controls, 69 heart failure survivors, 39 heart failure nonsurvivors) reveals consistent multifractal scaling patterns. The heatmaps (Figure 2, *top panels*) display coefficient of determination ($r^{2}$) values for each individual across moment orders q∈[−10,10], showing how scaling reliability spans the full spectrum from negative (weak fluctuations) to positive (strong fluctuations).

All three groups exhibit the same fundamental pattern: $r^{2}$ values are lower at negative moment orders (q<0), reach maximal values around q∈[−2,2], and gradually decline at positive moment orders (q>2). This consistent pattern—resembling additomultiplicative synthetic cascades rather than purely additive or multiplicative patterns (Figure 1)—confirms that cardiac regulation employs hybrid mechanisms combining additive and multiplicative operations across health and disease states.

The threshold analysis (Figure 2, *bottom panels*) shows similar expansion patterns across all groups. At strict thresholds ($r^{2}>0.95$), all groups show concentrated regions around q∈[−1,1]. As thresholds relax to $r^{2}>0.85$, valid *q*-ranges extend with slight rightward bias toward positive *q*. Comparing to synthetic cascade predictions (Figure 1): purely additive cascades would show complete breakdown of scaling relationships at negative *q* with total high $r^{2}$ leading to full yellow coverage across all positive *q*—instead, we observe gradual, moderate expansion toward positive *q* while maintaining valid scaling around q∈[−2,2]. Purely multiplicative cascades would show minimal expansion in any direction—not observed. The observed pattern of symmetric maintenance with gradual rightward expansion is the diagnostic signature of additomultiplicative organization, maintained consistently across healthy controls and heart failure patients. Notably, no systematic differences in threshold sensitivity patterns were observed across the three cardiac groups, indicating that heart failure does not fundamentally alter the additomultiplicative cascade structure.

These findings differ from predictions based solely on synthetic cascade comparisons, where additomultiplicative organization was characterized by symmetric threshold expansion. In cardiac data, all groups—healthy and pathological—exhibit similar threshold sensitivity patterns, suggesting that the fundamental additomultiplicative cascade structure is preserved across health and disease states. This preservation indicates that heart failure does not alter the underlying generative mechanism but may instead affect other aspects of multifractal organization, such as the operational range of reliable scaling (quantifiable through metrics like $N_{q}$) or the precision of individual scaling relationships [12]. The universal additomultiplicative signature across cardiac groups demonstrates remarkable structural stability despite varying degrees of autonomic dysfunction.

### 3.3. Financial Markets Maintain Additomultiplicative Consistency Across Asset Classes

**Figure 3:**
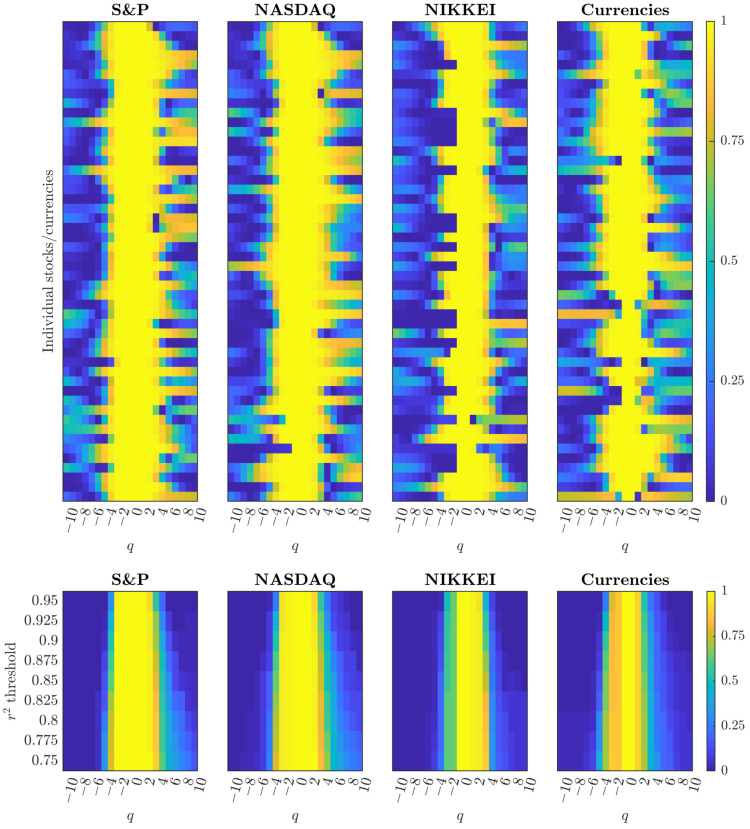
Financial markets exhibit additomultiplicative organization with asset-class-specific variations in scaling robustness. **Top:** $r^{2}$ heatmaps across moment orders q∈[−10,10] for S&P 500, NASDAQ, NIKKEI constituents, and major currency pairs (n=50 per group). Each row is a time series; each column is a *q*-value. Higher $r^{2}$ values (yellow) indicate stronger linear fits in the Chhabra–Jensen direct method [13]. S&P 500 and NASDAQ show robust central bands with rightward extension, NIKKEI displays moderate patterns, while currency pairs exhibit highly variable scaling reliability. **Bottom:** Proportion achieving each $r^{2}$ threshold reveals systematic differences in cascade robustness. S&P 500 and NASDAQ maintain high proportions across q∈[−4,3] even under strict thresholds, indicating precise additomultiplicative coupling. NIKKEI shows moderate threshold sensitivity, while currency pairs display dramatic expansion as thresholds relax, confirming additomultiplicative structure with greater noise potentially from 24-h trading, cross-border exposure, and diverse regulatory environments. All asset classes preserve the rightward-biased expansion characteristic of additomultiplicative organization, with variations reflecting market microstructure rather than fundamental cascade type.

Financial market analysis reveals systematic variations in multifractal scaling robustness across asset classes while maintaining consistent additomultiplicative signatures. The heatmap analysis (Figure 3, *top panels*) demonstrates that S&P 500 and NASDAQ constituents exhibit robust central bands of high $r^{2}$ values (yellow regions) with rightward extension toward positive *q*, similar to patterns observed in cardiac dynamics. NIKKEI constituents display moderately increased heterogeneity, while currency pairs show the most variable scaling reliability across individual time series and the full *q*-spectrum.

The threshold analysis (Figure 3, *bottom panels*) quantifies differences in cascade robustness across market types. At strict thresholds ($r^{2}>0.95$), S&P 500 and NASDAQ maintain high proportions achieving valid scaling across q∈[−4,3], indicating highly reliable multifractal organization. NIKKEI constituents show moderate threshold sensitivity, while currency pairs exhibit the greatest dependence on relaxed thresholds, with dramatic expansion as $r^{2}$ requirements decrease.

All asset classes show rightward-biased expansion under relaxed thresholds—extending more toward positive *q* (large price movements) than negative *q* (small adjustments)—confirming additomultiplicative organization across all financial systems. Quantitative differences in threshold sensitivity reflect varying organizational robustness rather than fundamental cascade type, with US equity markets demonstrating the most precise additomultiplicative coupling and currency pairs showing the same structure with greater noise potentially from 24-h trading, cross-border exposure, and diverse regulatory environments.

### 3.4. Climate Systems Exhibit Geographical Variations Reflecting Atmospheric Dynamics

**Figure 4:**
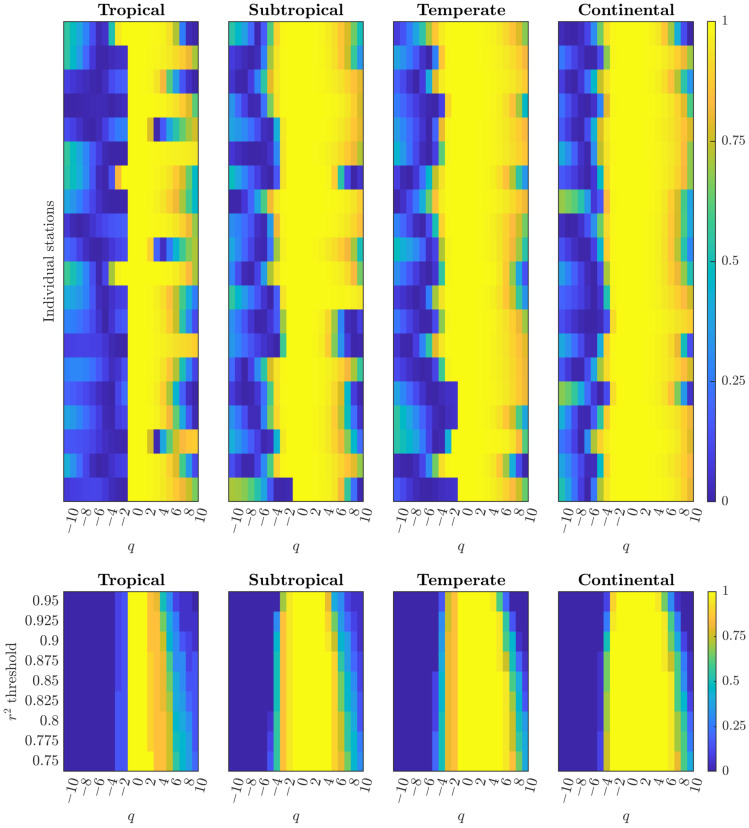
Climate systems exhibit additomultiplicative organization with geographical variations in scaling robustness. *Top:* $r^{2}$ heatmaps across moment orders q∈[−10,10] for tropical, subtropical, temperate, and continental climate zones (n=20 stations per zone). Each row is a station; each column is a *q*-value. Higher $r^{2}$ values (yellow) indicate stronger linear fits in the Chhabra–Jensen direct method [13]. Subtropical and continental zones show broad, stable high-$r^{2}$ bands across broad *q*-ranges, temperate zones display intermediate patterns, while tropical zones show more constrained scaling reliability. *Bottom:* Proportion achieving each $r^{2}$ threshold reveals systematic geographical differences in cascade robustness. Subtropical and continental zones maintain high proportions across q∈[−3,6] even at strict thresholds, indicating the most robust additomultiplicative organization. Temperate zones show slightly reduced robustness, while tropical zones exhibit the most constrained patterns requiring greater threshold relaxation. All climate zones preserve rightward-biased expansion characteristic of additomultiplicative organization, with variations reflecting atmospheric forcing complexity (synoptic activity in higher latitudes versus tropical stability) rather than fundamental cascade type.

Analysis of daily maximum temperature indicates systematic geographical variation in the robustness of multifractal scaling, while preserving consistent additomultiplicative signatures across regions. The heatmap analysis (Figure 4, *top panels*) demonstrates that subtropical and continental zones exhibit the most coherent $r^{2}$ patterns, with broad, stable bands of high scaling reliability (yellow regions) extending across broad ranges of moment orders. Temperate regions show comparable but slightly more variable $r^{2}$ distributions, while tropical regions display more constrained scaling concentrated primarily around moderate *q* values with patches at extreme positive *q*.

The threshold analysis (Figure 4, *bottom panels*) quantifies systematic differences in cascade robustness across climate zones. At strict thresholds ($r^{2}>0.95$), subtropical and continental climates maintain high proportions achieving valid scaling across q∈[−3,6], indicating the most robust additomultiplicative organization. Temperate zones exhibit slightly reduced robustness with somewhat narrower *q*-ranges, while tropical regions show the most constrained patterns, with lower proportions under strict constraints but substantial expansion as $r^{2}$ requirements relax to moderate levels ($r^{2}>0.85$).

All climate zones show rightward-biased expansion under relaxed thresholds—extending more toward positive *q* (extreme events) than negative *q* (daily fluctuations)—confirming additomultiplicative organization across all atmospheric systems. This pattern mirrors cardiac and financial dynamics, revealing a consistent principle across domains: baseline regulatory mechanisms (daily temperature cycles, parasympathetic control, incremental price adjustments) maintain tighter scaling precision than adaptive responses to perturbations (extreme weather events, sympathetic bursts, volatility spikes). Quantitative differences in threshold sensitivity reflect varying atmospheric forcing complexity—synoptic activity in higher latitudes generating robust organization versus tropical stability producing more constrained patterns—rather than fundamental differences in cascade type.

## 4. Discussion

We have established that additomultiplicative cascades—hybrid processes randomly alternating between additive stabilization and multiplicative amplification—universally govern multifractal scaling in complex systems. This conclusion rests on a critical methodological innovation: examining how scaling reliability patterns respond to systematically relaxed analytical constraints reveals underlying generative mechanisms that absolute reliability metrics alone cannot distinguish. Across cardiac dynamics, financial markets, and climate systems, we discovered the diagnostic signature of additomultiplicative organization: expansion of valid scaling behavior under relaxed $r^{2}$ thresholds, spanning both weak fluctuations (negative *q*) and strong fluctuations (positive *q*). This pattern persists despite quantitative variations in organizational robustness, confirming that environmental constraints modulate cascade dynamics while preserving fundamental hybrid structure.

### 4.1. Threshold Sensitivity Analysis: From Quality Control to Mechanism Discovery

Traditional multifractal analysis applies $r^{2}$ thresholds to ensure statistical reliability of scaling relationships, retaining only moment orders where linear regressions meet reliability criteria [14]. While this filtering approach ensures robust spectrum estimation, it treats all moment orders meeting the threshold as equivalent and discards those that fail, regardless of whether systematic patterns exist in the excluded regions. Our findings reveal that this binary accept/reject framework overlooks critical diagnostic information. The systematic variation in how scaling relationships degrade across the *q*-spectrum carries mechanistic information about the underlying generative processes.

Our reliance on $r^{2}$ as the primary diagnostic metric warrants methodological justification, particularly given its well-documented limitations, including sensitivity to outliers, inability to detect systematic departures from linearity, and dependence on the range of independent variables. However, these limitations are diagnostically informative for threshold sensitivity analysis. Our framework requires a *continuous measure* of scaling reliability that varies systematically across moment orders, enabling examination of how fit quality degrades from moderate to extreme *q* values. The $r^{2}$ coefficient provides exactly this property: it quantifies the proportion of variance explained by the power-law scaling model at each *q*, with degradation patterns (symmetric, asymmetric, or constrained) distinguishing cascade types. Hypothesis testing approaches (e.g., testing whether regression slopes differ significantly from zero) would provide only binary accept/reject decisions at each *q*, discarding the graded reliability information central to our diagnostic framework. Alternative measures, such as the Akaike Information Criterion, require model comparison frameworks inappropriate for power-law scaling, where the functional form is theoretically specified rather than empirically selected. The systematic variation in $r^{2}$ across the *q*-spectrum reveals how different cascade mechanisms produce characteristic patterns of fit degradation—precisely the diagnostic signature we seek to identify.

The diagnostic information lies not in which moment orders meet arbitrary thresholds, but in how the valid *q*-range expands as constraints relax. Systems employing different generative mechanisms respond distinctively to this relaxation: additomultiplicative cascades show expansion across both weak and strong fluctuations, additive cascades show complete breakdown at negative *q* with total high $r^{2}$ across all positive *q*, and multiplicative cascades maintain narrow reliability zones regardless of threshold (Figure 1).

This methodological shift transforms $r^{2}\left(q\right)$ profiling from quality control into mechanistic classification. Cardiac dynamics (Figure 2) demonstrate consistent additomultiplicative organization across health and disease states. All groups show concentrated high-$r^{2}$ values around moderate moment orders with slight rightward expansion as thresholds relax. This pattern—moderate, gradual expansion toward positive *q* while maintaining validity at negative *q*—distinguishes additomultiplicative from purely additive organization (which would show complete breakdown at negative *q* with full yellow coverage at positive *q*) or purely multiplicative organization (which would show minimal expansion).

Market microstructure shapes cascade organization systematically across asset classes (Figure 3). S&P 500 and NASDAQ show robust central bands of high $r^{2}$ values with rightward extension toward positive *q*, indicating tight scaling for large price movements. The proportion plots reveal precise additomultiplicative coupling with minimal threshold sensitivity. Currency pairs show dramatically different patterns: under strict constraints, valid scaling concentrates around moderate *q* in low proportions, but as thresholds relax, expansion emerges toward both negative and positive *q*. This confirms additomultiplicative structure with greater contribution of unstructured noise potentially from 24-h trading [33,34], cross-border exposure [35,36], and diverse central bank policies [37].

All climate zones exhibit additomultiplicative organization with rightward-biased threshold expansion (Figure 4). This pattern—where expansion occurs more toward positive *q* (extreme events) than negative *q* (daily fluctuations)—mirrors cardiac dynamics and mature financial markets. Geographical variations reflect differences in atmospheric forcing complexity (synoptic activity in higher latitudes versus tropical stability), not differences in fundamental cascade type [23,24,25,26,27].

The convergence of threshold sensitivity patterns across these three fundamentally different physical processes—cardiac autonomic control, financial price discovery, atmospheric energy cascades—establishes additomultiplicative organization as a universal principle while revealing that environmental constraints modulate organizational robustness rather than cascade type.

### 4.2. Resolving the Additive-Multiplicative Dichotomy

For four decades, cascade modeling has been shaped by an implicit binary, with structured variability largely attributed to multiplicative cascades and additive processes treated as null or noise models [38,39,40,41]. This either-or framework forced complex systems into artificially simplified categories, with debates centering on which single mechanism best explains observed multifractal scaling. Our empirical validation across three fundamentally different physical domains—cardiac, financial, climate—demonstrates that this dichotomy is false. ALL complex adaptive systems employ hybrid additomultiplicative mechanisms. This finding reframes how we model physiological regulation, market dynamics, and climate systems. The question is no longer which mechanism dominates, but how environmental constraints shape the expression of universal hybrid organization.

Real-world complex systems universally employ hybrid additomultiplicative mechanisms. Additive and multiplicative processes are not competing explanations but complementary components working in concert. Random alternation between additive operations (maintaining baseline variability, providing robustness) and multiplicative operations (enabling adaptive amplification, facilitating cross-scale information transmission) produces the diagnostic signature we observe: threshold expansion patterns with scaling reliability across both weak fluctuations (small, frequent variations) and strong fluctuations (large, rare events). This hybrid organization characterizes cardiac regulation across health and disease states, market price discovery across asset classes, and atmospheric dynamics across all climate zones examined.

The conceptual shift from binary classification to hybrid integration reframes the field. The question is no longer “additive or multiplicative?” but rather “how robustly is the additomultiplicative organization expressed?” Our findings reveal that environmental constraints and domain-specific dynamics modulate the mathematical bounds within which additomultiplicative dynamics operate, without altering the fundamental cascade type. Cardiac dynamics maintain consistent threshold patterns across health and disease states, indicating preserved additomultiplicative organization regardless of pathological status. Financial markets exhibit threshold signatures across all asset classes, with US equities showing tighter organization than currencies but both maintaining hybrid structure. Climate systems show varying robustness across geographical zones, with subtropical and continental regions exhibiting stronger scaling than tropical regions, while all maintain the same additomultiplicative pattern. This reconciles theoretical predictions of universal scaling with empirical observations of quantitative variation—the universality lies in the hybrid additomultiplicative mechanism, while the variation reflects domain-specific constraints that modulate organizational robustness within this fundamental framework.

### 4.3. Universal Principles and Critical Organization

The convergent findings across cardiac, financial, and climate systems provide compelling evidence for the universality of additomultiplicative cascade organization in complex adaptive systems. All three domains exhibit the characteristic symmetric expansion of valid scaling behavior under relaxed $r^{2}$ thresholds, spanning both weak fluctuations (negative *q* values) and strong fluctuations (positive *q* values), confirming that hybrid generative mechanisms maintain scale-invariant structure across the full spectrum of variability intensities and represent organizational principles rather than domain-specific phenomena. The quantitative variations in cascade robustness—observed across different financial markets (US equities showing tighter organization than currencies) and climate zones (subtropical/continental stronger than tropical)—reflect differences in environmental constraints and regulatory precision rather than fundamental differences in cascade type. Cardiac dynamics, notably, maintain consistent additomultiplicative signatures across health and disease states. This universal framework establishes $r^{2}$ threshold analysis with the Chhabra–Jensen direct method [13] as a powerful diagnostic approach for identifying and characterizing the generative mechanisms underlying multifractal scaling across diverse complex systems.

Crucially, these systems exhibit regulated rather than self-organized criticality [42,43,44]. Self-organized criticality predicts passive evolution toward critical states without external tuning. Our findings show something fundamentally different: additomultiplicative organization persists across cardiac health states, financial volatility regimes, and diverse climate zones. This persistence despite perturbations demonstrates active maintenance of critical organization through feedback mechanisms. Market stress increases noise but maintains a hybrid price discovery structure. Climate forcing modulates robustness but preserves additomultiplicative atmospheric organization. Cardiac dynamics maintain consistent threshold signatures regardless of disease state. This extends recent work on controlled criticality in neural systems [45,46,47], suggesting that additomultiplicative organization represents a general strategy for achieving regulated criticality across diverse physical domains—systems actively maintain the balance between additive and multiplicative processes rather than passively settling into critical states.

The universality of additomultiplicative organization across fundamentally different physical processes suggests a deep principle governing how complex systems achieve robust multiscale coordination. Random alternation between additive and multiplicative operations at local scales enables bidirectional information flow across hierarchical structures: additive components propagate baseline variability upward through linear superposition, maintaining coherent correlations across scales, while multiplicative components amplify perturbations downward through proportional scaling, enabling adaptive responses to propagate from coarse to fine scales. This hybrid architecture solves a fundamental tension in self-organizing systems—how to maintain stable baseline function while preserving capacity for rapid adaptation—by decoupling these functions across distinct mathematical operations that interact stochastically rather than deterministically. The resulting structure exhibits emergent robustness: neither operation alone can sustain multifractal scaling across fluctuation intensities, yet their random combination produces a scale-invariant organization that persists despite environmental perturbations, noise variations, and pathological degradation. This suggests that additomultiplicative dynamics may represent a general solution to the problem of maintaining organized complexity in the presence of uncertainty, applicable wherever systems must balance regulatory precision with adaptive flexibility across multiple spatial and temporal scales.

### 4.4. Limitations and Future Directions

Our analysis focused on systems with documented multifractal scaling across three specific domains. Several important limitations and extensions merit consideration. First, the framework requires validation on systems with unknown multifractal properties to establish its diagnostic utility beyond confirmed cases. Second, we examined threshold sensitivity under natural variation (health vs. disease, different markets, climate zones) but did not investigate responses to controlled external perturbations or longitudinal tracking of individual systems through transitions. Third, we analyzed univariate time series; extension to multivariate systems and higher-dimensional cascades represents an important frontier for understanding coupled dynamics across multiple channels or spatial dimensions. Fourth, while we demonstrated that threshold sensitivity patterns qualitatively distinguish cascade types through visual inspection of $r^{2}\left(q\right)$ heatmaps and proportion plots, we have not established quantitative classification metrics for objectively assigning empirical systems to cascade classes. Traditional scalar measures like operational range ($N_{q}$, counting valid moment orders) provide useful summary statistics but collapse the rich two-dimensional $r^{2}\left(q\right)$ signature into a single number, discarding diagnostic information about asymmetry, expansion patterns, and threshold sensitivity that distinguish cascade types. Developing rigorous quantitative methods—such as machine learning classifiers trained on synthetic cascade signatures, distance metrics in $r^{2}\left(q\right)$-space, or information-theoretic measures of pattern similarity—represents an important future direction. Such methods would enable automated, objective cascade classification and facilitate large-scale screening applications where visual inspection becomes impractical.

A well-established limitation in multifractal analysis is the increasing unreliability of scaling exponents at large |q|, arising from the dominance of increasingly rare events [48]. For negative *q*, the estimator becomes sensitive to the smallest fluctuations in the data, which may be dominated by measurement noise or numerical precision limits. For positive *q*, the estimator emphasizes the largest fluctuations, which are sparsest and therefore subject to substantial sampling variability—particularly in finite-length series. This issue affects all multifractal methods, not just the Chhabra–Jensen direct method [13]. The operational range of reliable moment orders depends on three interrelated factors: (*i*) series length, (*ii*) the thickness of the fluctuation distribution tails, and (*iii*) the specific estimator used. In our analysis, cardiac recordings were standardized to $N=2^{14}=16,384$ data points, enabling 11 scale levels, while financial and climate time series span 25 years with mean lengths exceeding N=5800 days, providing 12–13 scale levels. These lengths substantially exceed the $N\geq 2^{12}$ minimum recommended for reliable multifractal estimation. We acknowledge that our *q*-range (q∈[−10,10]) may exceed the domain of stable estimation for some cascade types—particularly multiplicative cascades, which generate the heaviest tails and exhibit the poorest $r^{2}\left(q\right)$ at extreme *q*. However, this is not a limitation but rather a diagnostic feature: the breakdown of scaling reliability at large |q| is itself informative about cascade structure. Multiplicative cascades fail at extremes precisely because they generate unbounded amplification without stabilization, while additomultiplicative cascades succeed because their hybrid structure constrains tail behavior while preserving heterogeneity. Critically, our conclusions rest on *comparative patterns* of threshold sensitivity rather than absolute parameter values. The diagnostic signature of additomultiplicative organization—symmetric expansion of valid scaling under relaxed $r^{2}$ thresholds across both negative and positive *q*—emerges consistently across all three domains despite varying data lengths. While increasing series length would reduce finite-size statistical fluctuations and smooth partition-function regressions, such smoothing reflects improved numerical stability rather than qualitative changes in the cascade mechanism. The moment-order-specific reliability patterns that distinguish cascade types persist across our data lengths, indicating they reflect intrinsic cascade organization rather than finite-size artifacts. Future work examining how the reliable *q*-range scales with series length and tail thickness would establish optimal analytical protocols, though such extensive parameter sweeps lie beyond the objectives and scope of the present comparative analysis under fixed, controlled conditions.

Financial time series spanning 25 years inevitably exhibit nonstationarity arising from regime shifts (bull/bear market transitions), regulatory changes (implementation of circuit breakers, high-frequency trading regulations), technological evolution (algorithmic trading, decimalization), and macroeconomic transitions (policy rate cycles, financial crises). Climate data similarly contain nonstationarities from long-term warming trends, decadal oscillations, and urbanization effects. Traditional time series methods require stationarity for reliable parameter estimation, but multifractal analysis was developed explicitly to characterize scale-invariant properties of nonstationary systems with time-varying statistics and heterogeneous dynamics [1,2,3,4,5,6,7]. Our threshold sensitivity analysis extends this framework by examining *patterns* of scaling reliability rather than absolute parameter values. The diagnostic signature of additomultiplicative organization—symmetric expansion of valid *q*-ranges under relaxed thresholds—reflects the cascade mechanism operating across the full temporal span, capturing how systems maintain hybrid organizational structure despite regime transitions. Critically, nonstationarity would manifest as irregular, inconsistent threshold patterns across individual realizations if it fundamentally disrupted cascade organization. Instead, we observe systematic, reproducible patterns within each domain: all financial asset classes show rightward-biased symmetric expansion, all cardiac groups maintain similar threshold profiles, and all climate zones preserve additomultiplicative signatures. This consistency indicates that nonstationarity modulates organizational robustness (e.g., currencies showing greater variability than US equities, tropical climates showing reduced robustness compared to continental zones) without altering fundamental cascade type. Future work could explicitly examine the temporal evolution of threshold sensitivity patterns by analyzing rolling windows or before/after comparisons around known regime shifts (e.g., 2008 financial crisis, COVID-19 pandemic), testing whether cascade organization remains stable or undergoes systematic transitions during extreme events. Such analysis would establish whether additomultiplicative structure represents a persistent attractor state that systems maintain across perturbations, or whether certain disruptions can induce transitions between cascade types.

These methodological considerations naturally lead to critical open questions with direct practical implications that remain unanswered: can $r^{2}$ threshold analysis detect early pathological transitions before conventional metrics indicate dysfunction, enabling earlier clinical intervention? How do therapeutic interventions (e.g., beta-blockers, exercise training) or regulatory changes (e.g., circuit breakers, capital controls) affect cascade precision, and can this inform treatment optimization? Do threshold patterns change predictably during system evolution or environmental transitions, providing advance warning of regime shifts? Can real-time monitoring systems based on threshold sensitivity provide actionable early warnings in clinical (autonomic dysfunction), financial (market instability), or climate (extreme event risk) contexts?

Beyond these practical applications, fundamental theoretical questions emerge from our findings. While cardiac dynamics maintain consistent additomultiplicative signatures across health and disease states, financial and climate systems show variations in organizational robustness. What determines these domain-specific differences in threshold sensitivity patterns? Can the degree of threshold robustness predict system resilience or proximity to catastrophic failure? The regulated criticality we observe—systems actively maintaining additomultiplicative organization despite perturbations—suggests feedback mechanisms operate across all three domains. What is the minimal control architecture required to sustain a regulated additomultiplicative organization? How do feedback mechanisms differ between biological (neural–mechanical coupling), economic (agent interactions), and physical (thermodynamic) systems while producing equivalent threshold signatures?

Finally, the symmetric expansion pattern raises questions about cascade microstructure: does the random alternation between additive and multiplicative operations occur at fixed probability (50-50) or do systems modulate this probability to adapt to environmental demands? Can threshold sensitivity analysis distinguish between different switching probabilities, revealing how systems tune their balance between stability and adaptability? Do correlations exist between successive operations (memory effects), or is the alternation truly random? These questions connect additomultiplicative organization to broader issues of adaptation and the emergence of complexity in physical systems. 

## Data Availability

The datasets used in this study can be obtained from the corresponding author, Madhur Mangalam (mmangalam@unomaha.edu), upon reasonable request.
